# Intergenerational connections through technology: Insights from the Technology Use in Later Life multi-site study

**DOI:** 10.1093/geroni/igaa057.3405

**Published:** 2020-12-16

**Authors:** Shannon Freeman, Hannah R Marston, Charles Musselwhite, Janna Olynick, Rebecca Genoe, Cory Kulczycki, Beibei Xiong

**Affiliations:** 1 University of Northern British Columbia, Prince George, British Columbia, Canada; 2 Open University UK, Milton Keynes, England, United Kingdom; 3 Swansea University, Swansea, Wales, United Kingdom; 4 University of Regina, Regina, Saskatchewan, Canada

## Abstract

With enhanced challenges to maintain social connections especially during times of social distancing due to the COVID-19 pandemic, the need for technology solutions grow. Technologies have become interwoven into the daily lives for many older adults. The Technology Use in Later Life (TILL) study investigated how the perceptions and use of technology both can foster new and leverage existing intergenerational relationships. Through a mixed methods study engaging older adults aged 70 years of age and greater across rural and urban sites in Canada and the UK (N=37), participants described how the interconnection between technology and intergenerational relationships was an integral component to social connectedness with others. Through a qualitative descriptive approach, it was noted that older adults leveraged intergenerational relationships with family and friends to adjust to new technologies and to remain connected to adult children and grandchildren especially when there is high geographic separation between them. Especially during times of COVID-19, younger family members can play an important role to introduce and teach older adults how to use, technologies such as digital devices, computers, and social networking sites. Participants emphasized the benefits of intergenerational connections to adopt and use technology in later life noting flexibility and willingness to overcome barriers to technology adoption and remain connected across the generations. The adoption and uptake of technologies may continue as viable options during times of social distancing to support older persons to remain independent, age in place, in both age-friendly cities and across rural geographies during and post COVID-19.

